# Bacterial colonization and extinction on marine aggregates: stochastic model of species presence and abundance

**DOI:** 10.1002/ece3.789

**Published:** 2013-10-03

**Authors:** Andrew M Kramer, M Maille Lyons, Fred C Dobbs, John M Drake

**Affiliations:** 1 Odum School of Ecology, University of Georgia140 E. Green St., Athens, Georgia, 30602; 2 Department of Oceans, Earth and Atmospheric Sciences, Old Dominion University4600 Elkhorn Avenue, Norfolk, Virginia, 23529

**Keywords:** Aquatic pathogens, attached bacteria, microbial population dynamics, organic aggregates, stochastic extinction, waterborne disease

## Abstract

Organic aggregates provide a favorable habitat for aquatic microbes, are efficiently filtered by shellfish, and may play a major role in the dynamics of aquatic pathogens. Quantifying this role requires understanding how pathogen abundance in the water and aggregate size interact to determine the presence and abundance of pathogen cells on individual aggregates. We build upon current understanding of the dynamics of bacteria and bacterial grazers on aggregates to develop a model for the dynamics of a bacterial pathogen species. The model accounts for the importance of stochasticity and the balance between colonization and extinction. Simulation results suggest that while colonization increases linearly with background density and aggregate size, extinction rates are expected to be nonlinear on small aggregates in a low background density of the pathogen. Under these conditions, we predict lower probabilities of pathogen presence and reduced abundance on aggregates compared with predictions based solely on colonization. These results suggest that the importance of aggregates to the dynamics of aquatic bacterial pathogens may be dependent on the interaction between aggregate size and background pathogen density, and that these interactions are strongly influenced by ecological interactions and pathogen traits. The model provides testable predictions and can be a useful tool for exploring how species-specific differences in pathogen traits may alter the effect of aggregates on disease transmission.

## Introduction

**Figure 1:**
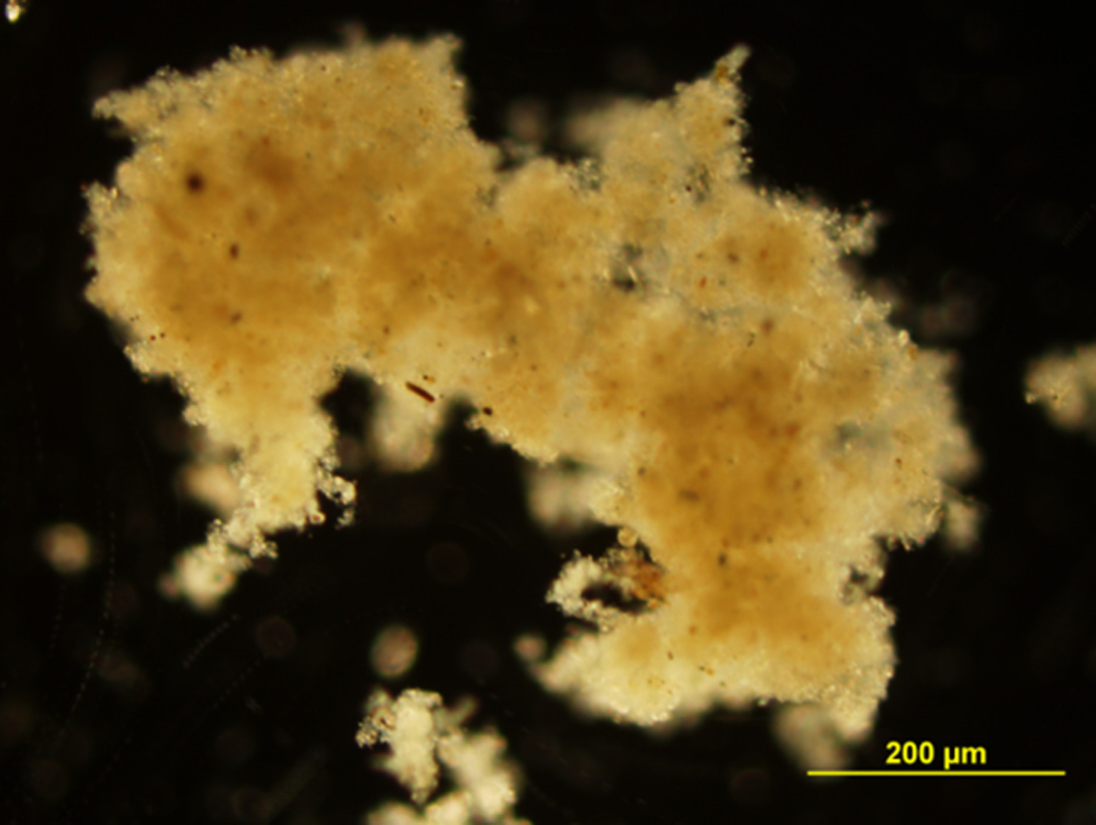
Organic marine aggregate formed in the laboratory using coastal seawater. Photo: M. Maille Lyons.

Organic aggregates, the clumps of material sometimes referred to as marine snow and bioflocs, are a crucial component of microbial dynamics in marine and freshwater ecosystems (Fig. 1; Caron et al. 1986; Alldredge and Silver 1988; Grossart and Simon 1998; Simon et al. 2002). They provide a productive substrate for a diverse microbial community and constitute a transport mechanism that can alternately remove attached microbes from the water column (Kiørboe 2001) or lead to resuspension from the sediments (Ritzrau and Graf 1992). Aggregates can also provide for increased ingestion of aquatic bacteria by suspension feeders such as oysters and clams, because capture efficiency of aggregates exceeds that of single cells in suspension (Riisgård 1988; Kach and Ward 2008). The role of aggregates as both favorable habitat and vectors for bacterial transport suggests they may play multiple roles in aquatic microbial dynamics.

Understanding some of these potential roles requires considering dynamics of individual microbial species on aggregates. For example, recent findings of several pathogenic and indicator bacteria incorporated into aggregates, including *Vibrio cholerae* (Alam et al. 2006), *V. vulnificus* (Froelich et al. 2013), *E. coli* (Lyons et al. 2010), and others (Lyons et al. 2007), suggest aggregates can influence pathogen abundance by offering favorable habitat for reproduction and survival. Despite species differences, the process of colonization and persistence on aggregates necessarily involve common physical and ecological interactions that should depend on species abundance in the water and aggregate characteristics. As a result, mechanistic modeling of microbe–aggregate interactions is a promising tool for providing quantitative estimates of the potential role of aggregates and guiding species-specific empirical investigation. Further, by providing a link between measurable environmental conditions such as pathogen density in the water and aggregate size distribution, a model can inform surveillance and risk assessments following events such as storms or release of contaminated water.

Developing a general model of how aggregates affect the distribution and abundance of pathogenic bacteria in aquatic ecosystems depends on understanding how species abundance in the water (“background density”) corresponds to presence and density of the species on aggregates. If the aggregate is considered to be an “island” in a sea of potential colonists, then the theory of island biogeography (MacArthur and Wilson 1967) suggests that bacterial colonization and extinction rates will be largely dependent on the size of the aggregate and its distance from the source of dispersing organisms – in this case the background density and relative abundance of bacterial species in the water (Bell et al. 2005; Lyons et al. 2010). The processes of colonization and extinction are stochastic, so individual species are expected to turn over while the overall species richness approaches an equilibrium (MacArthur and Wilson 1967; Whittaker 2000). As a result, accurately predicting the probability of occupancy for a target pathogenic species and its expected density on aggregates requires modeling these processes stochastically. This is particularly true when colonization rates are low or extinction rates are high, as is expected for small aggregates or for a species occurring at low densities, such as a pathogen at a distance from a point source.

Developing a plausible model for the dynamics of individual species on an aggregate is possible because of previous research. A mechanistic model designed to capture the dynamics of the entire bacterial community has been developed and validated by empirical data (Kiørboe 2003; Kiørboe et al. 2003). These authors found colonization rate to be determined by random motion of microorganisms and physical processes such as the sinking rate of particles with a contribution from bacterial swimming (Kiørboe et al. 2002; Grossart et al. 2003b). Colonization therefore depends on aggregate size and background bacterial density. This deterministic model has been found to describe well the abundance and dynamics of the entire community on the aggregate in terms of density-dependent growth rates, detachment and permanent attachment, and predation from higher trophic levels (Kiørboe et al. 2002, 2003; Kiørboe 2003), given the large number of individuals in the entire community. But individual species occur at lower densities and are subject to demographic stochasticity, which can result in qualitatively different dynamics and allow for extinction and recolonization, processes that cannot be assessed with the existing model. Therefore, understanding the dynamics of a particular species, such as a pathogen, requires understanding the dynamics of a multispecies community and considering the role of stochastic events. By incorporating these elements into the existing well-parameterized, empirically tested model of bacteria on aggregates, we aimed to understand how the balance between colonization and extinction controls presence and abundance of a target species, for example a pathogenic member of the overall bacterial community.

To do so, we developed a two-species, stochastic version of Kiørboe's (2003) model. Specifically, we explored the effect of aggregate size and the background density of a target species on its presence and abundance on aggregates. We assumed that the target species is indistinguishable from the overall bacterial community in its diffusion rate, reproduction, competition, and species interactions, and is present in the water as a subset of the total bacterial community. The model was then used to simulate the colonization–extinction balance due to demographic stochasticity. Presence and abundance of a target bacteria species was found to exhibit a threshold in its dependence on aggregate size and the density of the target species. The results provide qualitative and quantitative predictions of the percentage of aggregates carrying the target species, which we refer to as “occupancy,” and the pathogen load of individual aggregates for pathogen species with traits similar to the average aggregate-associated bacteria.

## Methods

We developed a multispecies, stochastic version of the mechanistic model for bacterial dynamics on marine aggregates proposed and parameterized by Kiørboe (2003) for the deterministic case. Our version models bacterial colonization, reproduction, predation, and detachment as a stochastic immigration–birth–death process (Renshaw 1993; Matis and Kiffe 2000). We focused on the density of a target bacterial species – which may be taken to represent a pathogenic species – on an individual aggregate also inhabited by a background community of bacterial species, flagellates grazing on bacteria, and ciliates preying on flagellates. Because detailed information concerning aggregate-related traits of specific pathogens is lacking, we made the basic assumption that the target species' traits and vital rates were equivalent to those of the background bacterial community. This assumption is helpful because it makes the model applicable to a range of species instead of a single pathogen, and is necessary because very little is known about the type of dynamics considered here for any individual bacterial species.

Deterministic equations describing population sizes of target bacteria (*E*), background bacteria (*B*), flagellates (*F*), and ciliates (*C*) follow Kiørboe (2003) and are extended by including two categories of bacteria, the general community and the target species. Further, bacterial populations are represented in two distinct possible states: committed, those which have adhered permanently to the aggregate, and uncommitted, those on the aggregate that may later detach back into the water (described in (Kiørboe 2003) text but not in the printed rate equation). These rates of change are as follows:


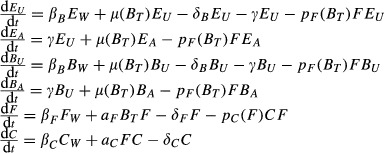


where *B*_*U*_ and *E*_*U*_ are uncommitted bacteria and target bacteria, *B*_*A*_ and *E*_*A*_ are committed bacteria and target bacteria; *B*_*T*_ is the sum of the bacterial community including committed, uncommitted, target, and background bacteria, that is, *B*_*T*_ = *B*_*U*_ + *B*_*A*_ + *E*_*U*_ + *E*_*A*_; *β*_*B*_, *β*_*F,*_ and *β*_*C*_ are “encounter rate kernels” normalized to account for encounter between the aggregate surface and organisms in the water; *δ*_*B*_, *δ*_*F*_, and *δ*_*C*_ are class-specific rates of detachment; and *γ* is the rate at which bacteria become irreversibly attached to the aggregate. The population growth rate of bacteria on the aggregate, *μ*, includes an effect of competition, with the relationship between growth rate and total bacterial density based on data from laboratory experiments with seawater (Kiørboe et al. 2003). Flagellate and ciliate growth rates, *a*_*F*_ and *a*_*C*_, are the product of predation rate (*p*_*F*_ and *p*_*C*_) and yields *Y*_*F*_ and *Y*_*C*_, respectively. Flagellate predation rate *p*_*F*_ depends on total bacterial density and ciliate predation rate *p*_*C*_ depends on flagellate density with both relationships described by a type-II function response (Holling 1966; Jürgens and Šimek 2000; Kiørboe et al. 2004):


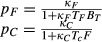


Following the standard theory for density-dependent prey consumption, *κ*_*F*_ and *κ*_*C*_ are the attack rates and *T*_*F*_ and *T*_*C*_ are the prey handling time for flagellates and ciliates, respectively (Holling 1959; Begon et al. 2006). The appropriateness of the saturating functional response is empirically supported (Fenchel 1980; Kiørboe et al. 2003).

This model was interpreted stochastically by taking the deterministic rates as the expected values of state transitions in an immigration–birth–death process (Renshaw 1993; Matis and Kiffe 2000). Thus, the probability of a particular transition in a short time interval Δt is the sum of the various mechanisms resulting in that transition (Table 1).

**Table 1:** Transition rates

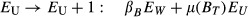
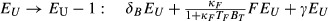
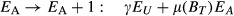
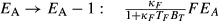
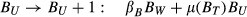
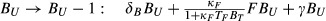
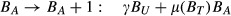
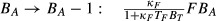
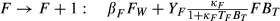
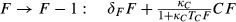
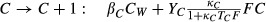
*C* → *C* − 1: *δ*_*C*_*C*

All parameters were constant across simulations, except target species density in the water and aggregate size. Kiørboe (2003) generated and collected default parameter values based on a combination of empirical and theoretical studies and found these parameter values were in general agreement with densities observed on actual aggregates. For clarity, the parameters we used are summarized in Table 2; we note that our notation differs slightly from that of Kiørboe (2003) in some subscripts in order to accommodate the target species and the committed and uncommitted classes of bacteria without confusion. Target species density in the water column was assumed to appear at one of four logarithmically distributed values (Table 2) and remain at that value throughout the simulation period. Aggregate radius was fixed between 0.01 and 1 cm (Table 2), with initial densities of the target species at zero and initial densities of bacteria, flagellates, and ciliates fixed at their deterministic expectation after 20,000 time steps. We remark that there is no inherent equilibrium because population size continues to increase slowly due to the permanent attachment process, but that this time frame ensures a well-established, resource-limited community to be present on the aggregate for the duration of the simulation. The dynamics of the target species on the aggregate were then simulated for ∼1 week (10,000 min), a similar time period as considered in previous empirical studies (Kiørboe et al. 2003, Kiørboe 2003, Froelich et al. 2013). Each combination of aggregate radius and target species density in the water was simulated from these initial conditions 960 or 1056 times (due to differences in architecture of computers used for simulation).

**Table 2:** Parameter values

Symbol	Definition	Units	Default Value^1^
*E*	Target bacteria density on aggregate	cells cm^−2^	–
*B*	Bacterial density on aggregate	cells cm^−2^	–
*C*	Ciliate density on aggregate	cells cm^−2^	–
*F*	Flagellate density on aggregate	cells cm^−2^	–
*r*	Aggregate radius	cm	0.01, 0.05, 0.1, 0.5, 1
*E*_*W*_	Background concentration of target bacteria	cells cm^−3^	10, 100, 1000, 10,000
*B*_*W*_	Background concentration of bacteria	cells cm^−3^	10^6^
*F*_*W*_	Background concentration of flagellates	cells cm^−3^	10^3^
*C*_*W*_	Background ciliate concentration	cells cm^−3^	10
*β*_*B*_	Encounter rate kernel for bacteria	cm min^−1^	Equation^2^
*β*_*F*_	Encounter rate kernel for flagellates	cm min^−1^	Equation^2^
*β*_*C*_	Encounter rate kernel for ciliates	cm min^−1^	Equation^2^
*μ*	Bacterial growth rate	min^−1^	Equation^3^
*δ*_*B*_	Bacterial detachment rate	min^−1^	2.3 × 10^−2^
*δ*_*F*_	Flagellate detachment rate	min^−1^	6.7 × 10^−3^
*δ*_*C*_	Ciliate detachment rate	min^−1^	6.4 × 10^−4^
*γ*	Permanent attachment rate of bacteria	min^−1^	2.3 × 10^−3^
*κ*_*F*_	Flagellate surface clearance	cm^2^ min^−1^	5 × 10^−7^
*κ*_*C*_	Ciliate surface clearance	cm^2^ min^−1^	1.25 × 10^−5^
*T*_*F*_	Flagellate prey handling time	min	0.24
*T*_*C*_	Ciliate prey handling time	min	0.24
*Y*_*F*_	Flagellate growth yield	cells cell^−1^	0.003
*Y*_*C*_	Ciliate growth yield	cells cell^−1^	0.003

1 Parameter values as in Kiørboe (2003) except for aggregate radius and background concentration of target bacteria.

2 Full expression and parameterization can be found in Eq. 2, Kiørboe (2003) or by requesting the code used here.

3 Full expression and parameterization can be found in Eq. 6, Kiørboe (2003) or by requesting the code used here.

Accurate simulation of small populations, such as the target bacteria at time zero, requires evaluating probabilities over very short time steps, but directly simulating each transition is computationally prohibitive because the large densities of background bacteria in the water and on the aggregate lead to correspondingly rapid transitions, preventing the simulation of long time periods. Although there are various approaches for simulating “stiff” systems like this one, we had success combining the binomial tau-leap (BTL) and optimized tau-leap (OTL) algorithms (available in the R package “GillespieSSA”; Pineda-Krch 2010; R Development Core Team 2011). Both algorithms use the current probabilities in the system to simulate multiple events in each longer step. OTL behaves by making larger leaps when stochasticity is less influential due to large population sizes and switching to single-step transitions when populations are small. For this problem, OTL was efficient and relatively accurate over most of the parameter space, but was supplemented using BTL for the first 10 min (in model time) of each simulation run. BTL uses a fixed step size designed to be small enough to maintain accuracy, but still be faster than the multitude of single steps that occurred most often during the first 10 min of simulated time. Actual computation time varied from hours to ∼1 week for each set of simulations of an aggregate community with a given parameter combination. All code is available upon request.

**Figure 2:**
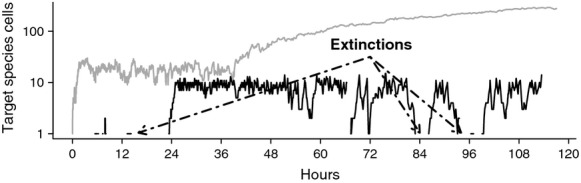
Representative trajectories from two parameter combinations. (1) aggregate radius *r* = 0.01 cm and background density of target species *E*_*W*_ = 1000 cells cm^−3^ (gray); and (2) *r* = 0.01 cm and *E*_*W*_ = 10 cells cm^−3^ (black). Arrows point out some of the extinction events at the lower *E*_*W*_. The population at higher *E*_*W*_ does not decline to extinction after initial colonization. The effect of tau-leap algorithms is seen in occasional changes of density exceeding 1 individual.

Information recorded from each simulation included every time point at which the target species colonized (transition from 0→1) or went extinct (transition from 1→0), the mean and maximum population size of target bacteria during the simulation, and the length of the simulation (Fig. 2). Simulation length was slightly longer than 10,000 min and varied due to the adaptive estimation of time steps. The information on time of colonization and extinction was used to calculate mean time to colonization, mean time to extinction, and proportion of time present. These data for each simulation were then combined to obtain means and 95% confidence range of the simulated realizations for each of 20 unique combinations of aggregate radius and density of target species in the water. These results were additionally generalized to order of magnitude expectations for the broader categories of rare versus common target species (background density ≤0.01% or 0.1–1% of the entire bacterial community, respectively) and small versus large aggregates (effective radius ≤0.1 or 0.5–1 cm.)

## Results

**Figure 3:**
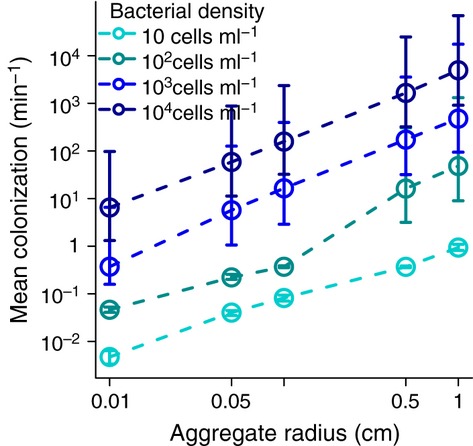
Mean time to colonization, the rate at which aggregates with no target species cells were colonized. Rates are the average of all colonization events over 10,000 simulated minutes; at high rates, this often corresponds to a single event. Axes are log_10_-transformed, and colors represent the constant background density of the target species in the water surrounding an aggregate. Means and 95% confidence intervals are for the rates from all simulations for each parameter combination.

Average rate of colonization, when an unoccupied aggregate gained an individual of the target species, increased monotonically with aggregate radius and the density of the target species in the water column (Fig. 3). The number of colonizations per minute increased consistently with both factors and a 10-fold increase in either had nearly the same impact on colonization rate, although radius had a slightly more complex effect via its two roles in determining the encounter kernel (Kiørboe 2003). Stochasticity had a more marked effect on variance at higher colonization rates, many of which experienced only one colonization event followed by persistence on the aggregate. Over the simulation (∼1 week), even the smallest aggregate in the presence of few target cells is very likely to become colonized.

**Figure 4:**
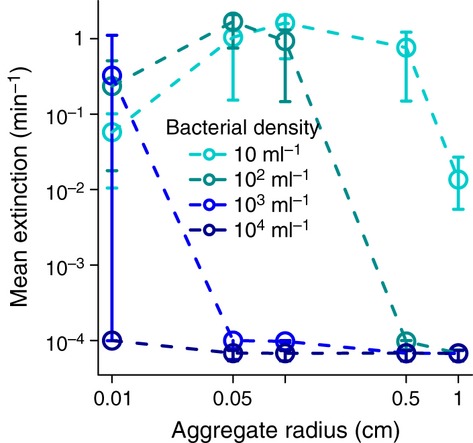
Mean time to extinction, the rate at which aggregates transition from hosting target species to a state with zero target species cells. Rates are the average of all extinction events over 10,000 simulated minutes; a rate of 10^−4^ indicates extinction did not occur following the initial colonization. Axes are log_10_-transformed, and colors represent the constant background density of the target species in the water surrounding an aggregate. Means and 95% confidence intervals are for the rates from all simulations for each parameter combination.

Extinction rate, by contrast, was very sensitive to aggregate size, except at the highest densities where the target species made up 1% (i.e., 10^4^ cm^−3^) of the bacterial cells in the water (Fig. 4). At low background densities, aggregates were likely to experience extinctions relatively rapidly following colonization, but this was offset by aggregate size for the two largest aggregates. The slowest rates of extinction essentially represent a lack of extinction over the simulation, as following the initial colonization, subsequent recruitment from the water column increased faster than the stochastic risk of extinction on the aggregate. The tendency of bacteria to become permanently committed also reduced extinction risk, because permanently attached bacteria are lost only due to predation, not the combination of predation and detachment. As a result, the extinction rate declined more with the addition of one committed bacteria than with the addition of one uncommitted bacteria, particularly when there were previously no committed bacteria. This created a discontinuity in extinction rates that had the most influence on extinction rates at intermediate probabilities of permanent attachment and contributed to a threshold in density and aggregate size after which extinction rate rapidly declined.

**Figure 5:**
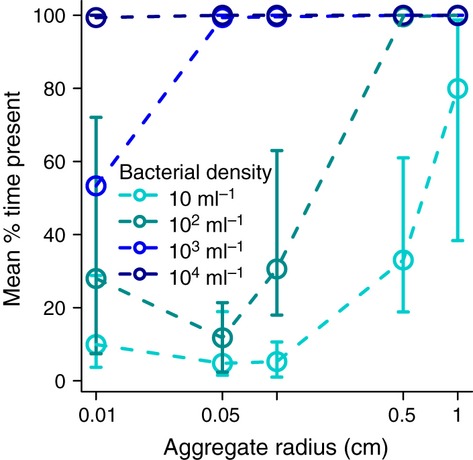
Presence of target species as a percent of simulation length. Colors represent the constant background density of the target species in water surrounding an aggregate and *x*-axis is log_10_-transformed. Means and 95% confidence intervals are for the percent of time present from all simulations for each parameter combination.

The target species occupancy on aggregates varied from <10% (on small aggregates at low background densities) to 100% (at high background densities; Fig. 5). We observed an unexpected threshold pattern for midsize aggregates at low densities, where the threshold observed in extinction rates translated into similar presence values across the small and midsize aggregates in low background densities of the target species followed by an abrupt increase in occupancy. This unanticipated pattern results from the interaction of changes in colonization, detachment and permanent attachment probabilities, and the stochastic loss of small populations. Even the smallest aggregates had a greater than 50% chance of containing the target species when background densities were high, but only large aggregates have a similar chance of containing the target species when background densities were low. Outside of the flat portion of the relationship, a doubling in aggregate size tended to more than double the probability that an aggregate will harbor the target species.

**Figure 6:**
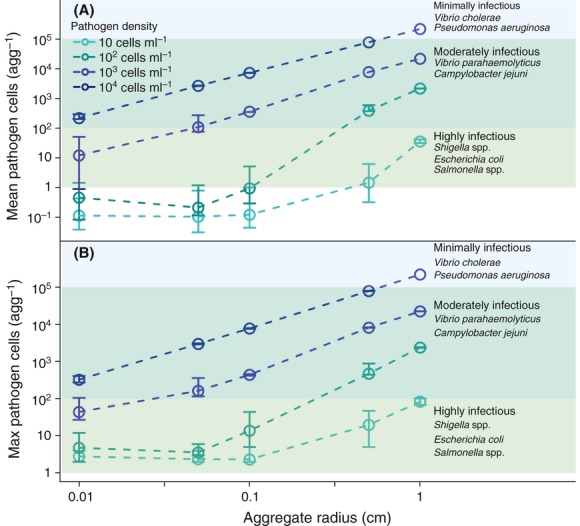
Population size of target species on aggregate. To illustrate how the theoretically obtained abundances relate to concentrations of pathogens relevant to human health (i.e., infectious doses), the population sizes are plotted with shaded regions representing aquatic bacterial pathogens with a corresponding minimum infectious dose. (A) Mean population size over the entire simulation. (B) Maximum population size over the simulation, usually corresponds to the end of the simulation. Axes are log_10_-transformed, and colors represent the constant background density of the target species in the water surrounding an aggregate. Means and 95% confidence intervals are for the population sizes from all simulations for each parameter combination. Minimum infective dose for a selection of waterborne bacterial pathogens (Rusin et al. 1997; Lampel et al. 2012).

The number of cells observed on aggregates displayed a similar pattern of unchanging abundance with aggregate size at low background densities, but increased monotonically at higher densities (Fig. 6). At low densities and aggregates ≤0.1 cm radius, average population of the target species over the week of simulated time was generally below 1 cell aggregate^−1^. Average population sizes reached as high as 10^5^ cells aggregate^−1^ for large aggregates at high background densities of the target species. The maximum number of cells was below 100 for small aggregates and aggregates at densities of 10 cells cm^−3^, but reached very high levels for large aggregates at high densities. The maximum cell numbers were nearly always observed at the end of the simulation due to the gradual increase in permanently attached cells. The observed dynamics resulted in high correspondence between mean and maximum target species abundance. To illustrate how these theoretically obtained abundances relate to concentrations of bacteria relevant to human health, we plotted infectious doses of several bacterial pathogens alongside (Fig. 6; Rusin et al. 1997; Lampel et al. 2012). In practical terms, these model-derived abundances ranged from at or below the minimum infectious dose for highly infective pathogens when background densities are very low or low with small aggregates, while other combinations resulted in single aggregates carrying minimum infectious doses for moderate or minimally infective pathogens.

## Discussion

This study used stochastic simulation of colonization and dynamics on aggregates to better characterize, relative to previous models, how aggregate size and background density affect dynamics of a target species such as a pathogen. The presence and abundance of a target bacterial species on organic aggregates varied with aggregate size and background density in a complex way. Although colonization increased monotonically with either aggregate size or background density of the target species, demographic stochasticity and the dynamics on the aggregate created a threshold in the relationship between these factors and presence on the aggregate. The expected density of the target species on the aggregate was also flat with respect to aggregate size and background density when colonization rates were low due to a combination of small aggregates (*r* < 0.1 cm) and low background species density (*E*_*W*_ ≤ 100 cells cm^3^). Small aggregates and low background density also result in much higher variance in presence and abundance.

As these aggregate sizes and background densities of the target species correspond to the natural range of these parameters (Grossart et al. 2003a), the model provides some insight into the fraction of sampled aggregates expected to carry bacterial pathogens and their abundance on aggregates. Across a wide range of small-sized aggregates in low pathogen density water, many aggregates will be pathogen-free and aggregates with pathogens will represent very low doses. Of course, direct contact or ingestion with aggregates is only one method of transmission, and this does not account for the possible accumulation of pathogens in seafood via cumulative consumption of a range of large and small aggregates. If pathogen densities are higher than 10^4^ cm^−3^, which represents 1% of the concentration of all species combined, water itself will contain very high doses (e.g., Worden et al. 2006), but the accumulation in seafood with the aggregate as a vector may be even more important due to higher uptake efficiencies for aggregates (Froelich et al. 2013).

The observed patterns in extinction rates are not independent of colonization rates in this system. If colonization happens quickly, then extinction will not be observed even if loss rates are high, for example if predation rates are higher than reproduction rates. When colonization rates were low due to a combination of small aggregates and low background densities, extinction rates were highly variable, and were flat, or even increased, with aggregate size before decreasing on the largest aggregates. Several processes are influencing this pattern, including increased predator density supported by the gradually increasing density of permanently attached bacteria and an increase in colonization and therefore potential extinctions.

The tendency of bacteria in natural assemblages to become permanently committed to the aggregate in less than 1 day (Kiørboe et al. 2003) appears to be crucial to the observed dynamics, particularly as it leads to ever-increasing bacterial (and therefore flagellate and ciliate) populations over an aggregate life span of 1 week. At the same time, it creates a discontinuity in the rate of extinction once the attached populations are established, because loss rates are much lower for those populations. This result has public health ramifications, as pathogens will tend to persist even after disappearing from the water column. Kiørboe et al. (2003) observed such permanent attachment in some bacterial strains but not others and chose the rate used here (10% of the detachment rate) to agree with empirical observations on agar spheres. Differences in detachment rate and tendency to permanently attach would result in different species displaying very different dynamics on aggregates.

These results depend on several key assumptions in addition to that of permanent attachment. The density of the target species in the water was assumed to be constant over the 1-week time frame of the simulation; orders of magnitude variation in this density would alter abundance and presence. However, the rates involved suggest that initial high densities will lead to persistent populations on the majority of aggregates as individuals are rapidly accumulated and then reproduce on the aggregates. This implies a second assumption that aggregates are good habitat where pathogens survive and reproduce, a condition strongly supported by empirical data (Venkateswaran et al. 1990; Alam et al. 2006; Lyons et al. 2007). Additionally, we assumed that the target pathogen traits are equivalent to the overall community for which parameters were originally estimated. The validity of this assumption is unknown. The chance that it is strictly true is clearly low, because species differ in crucial characteristics such as size, mobility, or substrate utilization, and even strains of the same species can have different interactions with aggregates (Froelich et al. 2013). However, assessing the direction and magnitude of differences for individual pathogen species constitutes a subject for further research. Importantly, it is unlikely that life history characteristics measured in isolation are directly relevant, as growth rate, detachment rate, and mortality rates in the model all integrate over the competition and predation taking place in a mixed community on aggregates. We note that additional species-specific interactions are likely, such as mortality due to phage (Riemann and Grossart 2008) and bacteria–bacteria inhibition (Grossart et al. 2004), and that such processes are here subsumed in shared rates of detachment, predation, and birth rate. A final assumption is that aggregate size is constant over the simulation, whereas natural aggregates can have a tendency to break up or accumulate additional material. The balance of these processes may result in a stable size structure that could be characterized by these results, but the consideration of aggregate physical dynamics was beyond the scope of this study.

These findings extend previous, generalized models and are useful to guide further research on the dynamics of water-borne pathogens. Particularly, our simulation results provide testable predictions that can be addressed by observations of natural aggregates and experiments. (1) Our model predicts that the relationship between the percent of aggregates found positive for a pathogen at low density in the water, and the size of the aggregates should be relatively flat for small aggregates and then increase sharply for larger aggregates, while aggregates in high background densities of pathogen should almost always contain pathogen cells. (2) Similarly, we predict that the abundance of a pathogen on aggregates will increase nearly linearly with either aggregate size or background density, except for small aggregates at low density. Field collection of a range of aggregate sizes from different background densities or laboratory formation of aggregates from water with manipulated pathogen densities would allow this prediction to be tested. (3) Our results also predict that older aggregates are more likely to have pathogens present and should have higher abundances than younger aggregates. This prediction would require sampling aggregates of known age, requiring laboratory formation of the aggregates. We are unaware of any existing quantitative observations addressing these predictions.

The results from this model suggest that bacterial species abundance on aggregates depends on an interaction between the size distribution of the aggregates and the concentration of a species in the water. At relevant aggregate sizes, any relatively rare species is only expected to appear on some aggregates and only at low density, despite being present in the water surrounding the aggregate. This pattern results from stochastic loss from detachment and consumption by predators outweighing growth and recruitment from the water. These theoretical results establish several qualitatively different patterns to act as a starting point for considering species-specific differences of important aquatic bacteria, such as human pathogens. The model can be a useful tool for assessing the importance of aggregates to infection rates of bacterial pathogens, particularly because sampling aggregate size and background water is easier than isolating and quantifying aggregate-associated bacteria. The next step is to examine differences between pathogenic bacteria and between pathogens and common aquatic bacteria in colonization rates, detachment rates, predation rates, and attachment rates, as the theoretical results suggest these differences have the potential to alter infection risk of shellfish and human hosts. Examination of pathogenic *Vibrio spp*. would be a promising direction as they are currently studied in the laboratory and field, are known to interact with aggregates, and can be readily identified.
